# Pneumococcal Conjugate Vaccine Protection against Coronavirus-Associated Pneumonia Hospitalization in Children Living with and without HIV

**DOI:** 10.1128/mBio.02347-20

**Published:** 2021-01-08

**Authors:** Marta C. Nunes, Clare L. Cutland, Keith P. Klugman, Shabir A. Madhi

**Affiliations:** a Medical Research Council: Vaccines and Infectious Diseases Analytics Research Unit, Faculty of Health Sciences, University of the Witwatersrand, Johannesburg, South Africa; b Department of Science and Technology/National Research Foundation: Vaccine Preventable Diseases, University of the Witwatersrand, Johannesburg, South Africa; Department of Veterinary Medicine

**Keywords:** coronaviruses, HIV, pneumonia, respiratory infections, PCV9, pneumococcal vaccine, pneumococcal conjugate vaccine, SARS-CoV-2, *Streptococcus pneumoniae*, human coronavirus, human immunodeficiency virus

## Abstract

In December 2019 a new coronavirus (CoV) emerged as a human pathogen, SARS-CoV-2. There are few data on human coronavirus infections among individuals living with HIV. In this study we probed the role of pneumococcal coinfections with seasonal CoVs among children living with and without HIV hospitalized for pneumonia. We also described the prevalence and clinical manifestations of these infections. A total of 39,836 children who participated in a randomized, double-blind, placebo-controlled clinical trial on the efficacy of a 9-valent pneumococcal conjugate vaccine (PCV9) were followed for lower respiratory tract infection hospitalizations until 2 years of age. Nasopharyngeal aspirates were collected at the time of hospitalization and were screened by PCR for four seasonal CoVs. The frequency of CoV-associated pneumonia was higher in children living with HIV (19.9%) than in those without HIV (7.6%, *P* < 0.001). Serial CoV infections were detected in children living with HIV. The case fatality risk among children with CoV-associated pneumonia was higher in those living with HIV (30.4%) than without HIV (2.9%, *P* = 0.001). C-reactive protein and procalcitonin levels were elevated in 36.8% (≥40 mg/liter) and 64.7% (≥0.5 ng/ml), respectively, of the fatal cases living with HIV. Among children without HIV, there was a 64.0% (95% CI: 22.9% to 83.2%) lower incidence of CoV-associated pneumonia hospitalizations among PCV9 recipients compared to placebo recipients. These data suggest that Streptococcus pneumoniae infections might have a role in the development of pneumonia associated with endemic CoVs, that PCV may prevent pediatric CoV-associated hospitalization, and that children living with HIV with CoV infections develop more severe outcomes.

## Abstract (precis)

SARS-CoV-2 may cause severe hospitalization, but little is known about the role of secondary bacterial infection in these severe cases, beyond the observation of high levels of reported inflammatory markers, associated with bacterial infection, such as procalcitonin. We did a secondary analysis of a double-blind randomized trial of pneumococcal conjugate vaccine (PCV) to examine its impact on human coronavirus (CoV) infections before the pandemic.

## INTRODUCTION

Lower respiratory tract infections (LRTI) in young children are a major cause of hospital admissions (1). Globally, a large proportion of pneumonia hospitalizations are attributed to respiratory viral infections, including 61.4% in the multisite, international case-control PERCH (Pneumonia Etiology Research for Child Health) study undertaken in seven low- and middle-income countries among children aged 1 to 59 months (2). Coronaviruses (CoVs) are enveloped, nonsegmented, positive-sense, single-stranded RNA viruses and are associated with various natural hosts. Four CoVs are currently endemic in the human population, CoV-OC43, CoV-229E, CoV-NL63, and CoV-HKU1. CoV-OC43 and CoV-229E were initially identified as causes of upper respiratory tract infections in the 1960s using classical culture methods (3, 4). More recently, advances in molecular diagnostics have resulted in the identification of CoV-NL63 (5) and CoV-HKU1 (6). Infections by these endemic CoVs may involve the upper respiratory tract and the gastrointestinal tract; infections range from being completely asymptomatic to causing mild, self-limiting disease and, less commonly, severe illness such as bronchitis and pneumonia (7). Interestingly, hospitalized children with CoV-associated LRTI more frequently had coinfections with respiratory syncytial virus (RSV) and a higher abundance of Haemophilus influenzae than children hospitalized for non-CoV-associated LRTI or asymptomatic healthy CoV carriers (8), suggesting that viral and bacterial coinfections might trigger severe CoV infections.

Three other CoVs have, however, caused outbreaks of deadly pneumonia in humans since the beginning of the 21st century. The severe acute respiratory syndrome coronavirus (SARS-CoV), emerging in 2002, was responsible for an epidemic that spread to five continents with an overall fatality rate of approximately 10% with no fatalities reported in children (5), the Middle East respiratory syndrome coronavirus (MERS-CoV) has caused recurrent outbreaks in humans since 2012 mostly in the Arabian Peninsula with a fatality rate of 20% to 40% in adults and about 6% in children (9, 10), and the recent SARS-CoV-2 that appeared first in China at the end of 2019 has since caused a global pandemic (11). Although progress is rapidly being made, no specific antiviral vaccines are available to prevent any human CoV infections. Understanding the epidemiology and morbidity associated with common human-adapted CoVs may assist in informing treatment and mitigation strategies for severe illness from novel CoVs of zoonotic origin, although similar viruses may undergo different selective pressures in order to maintain fitness and host adaptation (12), making viral transmission patterns across species and their interactions with other potential coinfecting or superinfecting pathogens hard to predict (13).

Due to impaired humoral and cell-mediated immunity, children living with HIV (more so if not on antiretroviral treatment) have an increased risk for severe illness and mortality from virus-associated LRTI, including those due to RSV and influenza virus (14, 15). There are, however, limited data on the role of other respiratory viruses, including CoVs in children hospitalized with pneumonia, and only a few studies with limited numbers specifically included participants living with HIV (16, 17). A study in the United States involving immunocompromised children, the majority of whom had hematologic malignancy or solid tumor, found that these children were at higher risk of CoV-associated LRTI that necessitated oxygen use than were nonimmunocompromised children. In that study, younger age, underlying pulmonary disorder, and the concomitant presence of RSV were also associated with severe CoV-associated LRTI (18).

The aim of this study was to determine the burden and clinical features of CoV infections in children living with and without HIV hospitalized for pneumonia. Furthermore, as we have previously done for other respiratory viruses (19–21), we probed, using a randomized, placebo-controlled trial of an investigational 9-valent pneumococcal conjugate vaccine (PCV9), whether PCV9 could prevent CoV-associated hospitalization and thereby infer that pneumococcal infection may contribute to hospitalization for CoV-associated pneumonia.

## RESULTS

During the follow-up period included in this analysis, from all the participants in the initial trial, 347 (162 PCV9 group and 185 placebo recipients) children living with HIV and 448 (202 PCV9 group and 246 placebo recipients) without HIV had at least one episode of pneumonia tested for CoV infection. Similar proportions of samples were tested from PCV9 recipients and placebo recipients (22). Of the tested children, 69 (19.9%) living with HIV and 34 (7.6%; *P* < 0.001) without HIV tested CoV PCR positive (Table 1).

**TABLE 1:** Prevalence of human coronavirus-associated pneumonia in children 1 to 23 months old living with and without HIV

	No. (%)			*P* value^*b*^
	Overall (*n* = 750)	Living with HIV (*n* = 347)	Living without HIV (*n* = 448)	
CoV-OC43	68 (8.6)	56 (16.1)	12 (3.7)	<0.001
CoV-NL63	22 (2.8)	8 (2.3)	14 (3.1)	0.48
CoV-HKU1	22 (2.8)	13 (3.8)	9 (2.0)	0.14
CoV-229E	1 (0.1)	0	1 (0.2)	0.99
At least one CoV infection^*a*^	103 (13.0)	69 (19.9)	34 (7.6)	<0.001

a Some children had coinfection by two CoVs, and some had multiple episodes of CoV-associated pneumonia.

b *P* values adjusted for study group, i.e., placebo or pneumococcal conjugate vaccine.

Five children living with HIV had the same virus detected for long periods, including three children with CoV-OC43 detected 32, 38, and 70 days apart; one child with CoV-HKU1 identified twice 74 days apart; and one child in whom CoV-OC43 was identified twice 219 days apart. One child living with HIV had three CoV-associated admissions; the child initially tested positive for CoV-OC43, then 5 months later CoV-NL63 was detected, and after 3 months CoV-OC43 was detected again. Six children living with HIV had two distinct CoVs identified at two different admissions.

When assessing the CoV-associated pneumonia episodes among children randomized to placebo, children living with HIV compared to those without HIV were more likely to have oxygen saturation of <90% and stay in hospital for longer than 2 days. The percentage of CoV-associated pneumonia episodes with CoVs detected as single viral infection was similar among placebo recipients living with HIV compared to those without HIV (Table 2).

**TABLE 2:** Demographic, clinical, and laboratory characteristics of children living with and without HIV hospitalized with coronavirus-associated pneumonia^*e*^

	Living with HIV		Living without HIV		*P* value^*a*^
	Placebo	PCV	Placebo	PCV	
Specimens tested	252	207	256	212	-
CoV-positive samples	**44**	**37**	**25**	**9**	-
CoV detected as single viral infections	17 (38.6)	10 (27.0)	8 (32.0)	4 (44.4)	0. 91^*b*^
Mean age in mo (SD)	8.9 (6.3)	9.5 (6.4)	11.7 (6.1)	10.4 (6.4)	0.10
Axillary temp ≥38°C	8 (42.1) [19]	4 (18.2) [22]	3 (23.1) [13]	0 [7]	0.47
Mean respiratory rate, breaths per minute (SD)	57.5 (14.9)	56.3 (13.2)	51.1 (12.6)	58.7 (14.1)	0.076
Cyanosis	9 (21.4) [42]	13 (35.1)	2 (8.0)	1 (11.1)	0.34
Oxygen saturation on room air <90%	24 (54.6)	16 (43.2)	5 (20.0)	3 (33.3)	**0.013**
Bronchial breathing	10 (22.7)	7 (18.9)	2 (8.0)	1 (11.1)	0.08
Hospital stay >2 days	32 (76.2) [42]	27 (73.0)	18 (75.0) [24]	5 (55.6)	0.54
History of fever	18 (41.9) [43]	15 (40.5)	11 (44.4)	3 (33.3)	0.66
History of vomiting	0 [42]	2 (5.4)	2 (8.0)	0	0.14^*d*^
History of seizures	17 (46.0) [37]	13 (41.9) [31]	9 (36.0)	2 (25.0) [8]	0.25
Alveolar consolidation on chest X-ray	10 (22.7)	7 (18.9)	2 (8.0)	1 (11.1)	0.08
C-reactive protein ≥40 mg/liter	15 (44.1) [34]	12 (40.0) [30]	7 (35.0) [20]	3 (42.9) [7]	0.19
Procalcitonin ≥2 ng/ml	6 (30.0) [20]	10 (40.0) [25]	5 (27.8) [18]	2 (33.3) [6]	0.49
Mean white cell count cells, 10^9^/liter (SD)	12.8 (6.0) [13]	11.6 (6.7) [11]	23.1 (15.8) [6]	12.6 (7.2) [2]	0.14
Bacterial infection^*c*^	2 (5.1) [39]	6 (16.7) [36]	1 (4)	0	0.98

a *P* values compare the placebo group between children living with and without HIV, adjusted for age at hospitalization, detection of previously tested viruses, and year of collection. Values in bold indicate statistically significant comparisons.

b *P* values compare the placebo group between children living with and without HIV, adjusted for age at hospitalization, and year of collection.

c Blood specimens were available for culture from 109 coronavirus-associated pneumonia episodes, 75 HIV infected and 34 HIV uninfected. Bacteria isolated from children living with HIV included Streptococcus pneumoniae (*n* = 2), Escherichia coli (*n* = 3), *Salmonella* sp. (*n* = 1), Streptococcus viridans (*n* = 1), and other *Streptococcus* (*n* = 1). Bacteria isolated from children without HIV included *Streptococcus viridans* (*n* = 1) and *Micrococcus* (*n* = 1).

d *P* value calculated by Fisher’s exact test.

e Results are presented as number of participants with a particular presentation and percentage in parentheses, unless otherwise indicated. Numbers in brackets are number of participants with available information if different from total. PCV, pneumococcal conjugate vaccine. SD, standard deviation.

Bacterial cultures were performed in 94.8% of CoV-associated pneumonia admissions. Streptococcus pneumoniae was isolated from blood specimens of two children living with HIV collected at the same admission at which CoV-NL63 was detected in nasopharyngeal aspirate (NPA), one PCV9 recipient (serotype 23F) and one placebo recipient (serotype 6A).

Considering children with CoV-associated pneumonia, those living with HIV had a higher case fatality risk (30.4%, *n* = 21) than children without HIV (2.9%, *n* = 1; *P* = 0.001). This was similar to the case fatality risks for children hospitalized for pneumonia in whom CoV infections were not detected (27.0% living with HIV versus 1.7% without HIV). The median age of the 21 children living with HIV who died was 3 months (range: 1 to 15 months) (Table 3). CoV-OC43 was the only virus detected in eight of the fatal cases. A coinfection of CoV-OC43 and CoV-NL63 was detected in a 2-month-old fatal case. Among children living with HIV, the case fatality risk in those with episodes where CoVs were the only viruses detected (32.1%) was similar to that in episodes with coinfections by CoVs and other viruses (22.6%; *P* = 0.35). Escherichia coli was isolated from blood of three children (all living with HIV), and Pneumocystis jirovecii infection was identified in 10 (62.5%) children with HIV. Seven (36.8%) of the children living with HIV who died had levels of C-reactive protein of ≥40 mg/liter, and 11 (64.7%) had procalcitonin levels of ≥0.5 ng/ml (Table 3).

**TABLE 3:** Characteristics of the 22 children who died during hospitalization with coronavirus-associated pneumonia

Age at hospitalization (mo)	Gender	Living with HIV	Diagnosis	Study group	Virus(es) detected^*a*^	Chest X-ray result	CRP (mg/liter)	PCT (ng/ml)	Bacteria isolated from blood	*P. jirovecii* infection
1	Male	Yes	Pneumonia	PCV	CoV-OC43 + influenza	Normal	270	14.9	No	Not tested
2	Female	Yes	Pneumonia	PCV	CoV-OC43 + hRV	Normal	7	0.1	No	Yes
2	Female	Yes	Pneumonia	PCV	CoV-OC43	Alveolar consolidation	14	0.2	No	Yes
2	Male	Yes	Pneumonia	PCV	CoV-OC43	Alveolar consolidation	1	0.2	No	Yes
3	Male	Yes	Pneumonia	PCV	CoV-OC43 + hBoV	Normal	1	4.4	No	No
4	Female	Yes	Pneumonia	PCV	CoV-OC43 + hBoV	Normal	127	1.2	No	Yes
4	Male	Yes	Pneumonia	PCV	CoV-OC43	Alveolar consolidation	3	Unknown	No	Yes
9	Male	Yes	Pneumonia	PCV	CoV-OC43	Unknown	2	8.8	No	No
10	Female	Yes	Pneumonia	PCV	CoV-OC43 + WUPyV + hRV + influenza	Unknown	210	174.9	Escherichia coli	Not tested
14	Male	Yes	Pneumonia	PCV	CoV-OC43 + KIPyV	Alveolar consolidation	126	2.4	Escherichia coli	Yes
2	Male	Yes	Pneumonia	Placebo	CoV-OC43 + CoV-NL63	Normal	92	6.2	No	Not tested
2	Male	Yes	Pneumonia	Placebo	CoV-OC43 + hRV	Normal	1	0.2	No	Yes
2	Female	Yes	Pneumonia	Placebo	CoV-OC43 + hRV	Unknown	6	0.1	No	Not tested
2	Male	Yes	Pneumonia	Placebo	CoV-OC43 + KIPyV	Alveolar consolidation	21	0.6	No	Yes
3	Male	Yes	Pneumonia	Placebo	CoV-OC43	Normal	2	0.1	Not performed	No
3	Female	Yes	Pneumonia	Placebo	CoV-OC43 + hRV	Alveolar consolidation	39	Unknown	No	No
5	Female	Yes	Pneumonia	Placebo	CoV-OC43	Unknown	Unknown	Unknown	Not performed	No
7	Female	Yes	Pneumonia	Placebo	CoV-OC43	Normal	Unknown	Unknown	No	Not tested
8	Female	Yes	Pneumonia	Placebo	CoV-OC43 + hBoV	Normal	218	302.0	Escherichia coli	Yes
11	Female	Yes	Pneumonia	Placebo	CoV-HKU1 + hRV + KIPyV + influenza	Alveolar consolidation	37	0.66	No	Yes
15	Female	Yes	Pneumonia	Placebo	CoV-OC43	Alveolar consolidation	464	254.7	No	No
3	Female	No	Pneumonia	Placebo	CoV-229E + KIPyV	Unknown	1	Unknown	No	Yes

a Including viruses detected by immunofluorescence assay (influenza virus, respiratory syncytial virus [RSV], parainfluenza viruses [PIV], and adenovirus), nested PCR (human metapneumovirus), and PCR (human bocavirus [hBoV]; human rhinovirus [hRV]; human coronavirus [CoV]-OC43, -NL63, -HKU1, and -229E; and polyomaviruses WU [WUPyV] and KI [KIPyV]). PCV, pneumococcal conjugate vaccine. CRP, C-reactive protein. PCT, procalcitonin.

Overall, PCV9 recipients had a 33.9% (95% CI: 2.0% to 55.4%) lower risk for CoV-associated pneumonia hospitalizations than placebo recipients. In children living without HIV, PCV9 vaccination was associated with a 64.0% (95% CI: 22.9% to 83.2%) reduction in hospitalization with CoV-associated pneumonia, including reductions of 90.9% (95% CI: 29.5% to 98.8%) and 87.5% (95% CI: 0.1% to 98.4%) for CoV-OC43- and CoV-HKU1-associated hospitalizations, respectively. No vaccine efficacy was observed for pneumonia episodes without viruses identified (Table 4). No significant protection of PCV9 against hospitalization for CoV-associated pneumonia was observed in children living with HIV (Table 4).

**TABLE 4:** Differences in incidence of coronavirus-associated pneumonia episodes between immunized children who received 9-valent pneumococcal conjugate vaccine and placebo recipients, intent-to-treat analysis

	All children				Children living without HIV				Children living with HIV			
	PCV^*c*^ (*n* = 19,922)	Placebo (*n* = 19,914)	Vaccine efficacy (95% CI)	*P* value	PCV (*n* = 18,633)	Placebo (*n* = 18,626)	Vaccine efficacy (95% CI)	*P* value	PCV (*n* = 1,289)	Placebo (*n* = 1,288)	Vaccine efficacy (95% CI)	*P* value
CoV-OC43	27 (0.1)	41 (0.2)	34.2 (−7.0, 59.5)	0.09	1 (0.01)	11 (0.06)	90.9 (29.5, 98.8)	0.004	26 (2.0)	30 (2.3)	13.4 (−45.6, 48.5)	0.60
CoV-NL63	12 (0.06)	10 (0.05)	−20.0 (−177.6, 48.1)	0.67	8 (0.04)	6 (0.03)	−33.3 (−284.1, 53.7)	0.59	4 (0.3)	4 (0.3)	0.08 (−2,986.7, 75.0)	0.99
CoV-HKU1	6 (0.03)	16 (0.08)	62.5 (4.2, 85.3)	0.033	1 (0.01)	8 (0.04)	87.5 (0.1, 98.4)	0.020	5 (0.4)	8 (0.6)	37.5 (−90.4, 79.5)	0.40
Any CoV^*a*^	41 (0.2)	62 (0.3)	33.9 (2.0, 55.4)	0.038	9 (0.05)	25 (0.1)	64.0 (22.9, 83.2)	0.006	32 (2.5)	37 (2.9)	13.6 (−37.8, 45.8)	0.54
CoV detected as single viral infections	14 (0.07)	23 (0.1)	39.2 (−18.2, 68.7)	0.14	4 (0.02)	8 (0.04)	50.0 (−66.0, 84.9)	0.25	10 (0.8)	15 (1.2)	33.4 (−47.7, 70.0)	0.31
All-cause pneumonia^*a*^	518 (2.6)	612 (3.1)	15.4 (5.0, 24.6)	0.004	318 (1.7)	369 (2.0)	13.9 (0.05, 25.7)	0.049	200 (15.5)	243 (18.9)	17.8 (2.4, 30.6)	0.024
Any virus-associated pneumonia^*b*^	304 (1.5)	389 (2.0)	21.9 (9.4, 32.7)	0.001	190 (1.0)	252 (1.4)	24.6 (9.1, 37.5)	0.003	114 (8.8)	114 (8.8)	16.9 (−5.3, 34.3)	0.13
Pneumonia without viruses identified	239 (1.2)	255 (1.3)	6.3 (−11.6, 21.4)	0.47	125 (0.7)	120 (0.6)	−4.1 (−33.7, 18.9)	0.75	114 (8.8)	135 (10.5)	15.6 (−6.9, 33.4)	0.16

a First episode: children with pneumonia episodes associated with different coronaviruses are counted in that virus for each first episode but only once in any coronavirus. Children with pneumonia episodes associated both with and without a virus are counted in that category for each first episode but only once in the total number of all-cause pneumonia.

b Includes all viruses investigated: influenza virus, respiratory syncytial virus, parainfluenza viruses, adenovirus, human metapneumovirus, human bocavirus, human rhinovirus, coronaviruses, and polyomaviruses WU and KI.

c PCV, pneumococcal conjugate vaccine.

## DISCUSSION

The results of this study suggest a possible interaction between endemic human CoV infection and Streptococcus pneumoniae in children without HIV (23). In the context of a double-blind placebo-randomized trial, we demonstrated that at least 34% of children hospitalized for CoV-associated pneumonia were also probably coinfected by Streptococcus pneumoniae which might have precipitated their hospitalization. This is supported by *in vitro* experiments that have shown that CoV-NL63 infection resulted in an increased adherence of Streptococcus pneumoniae to virus-infected epithelial cells (24). The imputed rate of coinfection with pneumococci in children with CoV-associated pneumonia from our study provides a conservative estimate of this possible interaction as only 9 pneumococcal serotypes were included in the vaccine and vaccine efficacy, even against vaccine-serotype pneumococcal pneumonia, is not 100%. The high calculated vaccine efficacy estimates for CoV-OC43- and CoV-HKU1-associated disease needs to be contextualized within the wide uncertainty bounds of this estimate. Furthermore, we report on the prevalence and clinical features of endemic CoV-associated pneumonia in children living with and without HIV. CoVs were detected among 19.9% of the hospitalized children living with HIV and in 7.6% of children without HIV. CoV-associated mortality was 30.4% in children living with HIV with 38.1% of these fatal cases having CoV-OC43 as the only virus identified; mortality rates did not differ between children with CoV detected as single infections and those with other viral coinfections.

Although our study was not designed to establish whether CoV infections caused more severe disease in children living with HIV, among children with CoV-associated hospitalization, those living with HIV were more likely to have longer duration of hospitalization and a higher case fatality rate than children without HIV, similarly to what was previously described for other viral infections (14, 19, 25). The increased morbidity and mortality in children living with HIV could have been, however, due to increased susceptibility to pneumococcal and nonpneumococcal coinfections, such as *P. jirovecii*, Escherichia coli, Haemophilus influenzae, or other viral infections (8, 18). Other respiratory viruses were, however, not found at higher rates in children living with HIV infected with CoV compared to those without HIV. The high rate of *P. jirovecii* detection in fatal cases in children living with HIV also suggests that CoVs may lead to secondary *P. jirovecii* activation. Nonetheless, the higher case fatality rate and the fact that all eight fatal cases with CoV as the only respiratory virus detected were children living with HIV suggest a possible association of CoV causing severe disease in these children.

In the current study, of the children living with HIV who died, 36.8% and 64.7% had high levels of C-reactive protein and procalcitonin, respectively. Elevated levels of C-reactive protein and procalcitonin are normally detected in patients with bacterial infections compared to those with viral infections alone (26). However, high levels of these two markers may also be suggestive of unrecognized bacterial coinfections in patients with established viral etiology for pneumonia (27). Recent reports have described clear associations for raised C-reactive protein and procalcitonin levels with severity in patients with SARS-CoV-2 infection (28), including in fatal SARS-CoV-2 confirmed cases (29), further underscoring the complexity of host-novel pathogen interaction. Our results together with other data from seasonal CoVs (8) and SARS-CoV-2 suggest a role in bacterial coinfection in those CoV-infected patients who develop severe forms of disease.

Recurrent CoV-associated pneumonia episodes were detected in children living with HIV. This could be the result of extended viral shedding since these children have compromised cell-mediated immunity, which is necessary for termination of viral shedding, or due to viral reactivation or reinfection. Nonetheless, in these children initial immune response to infection was not enough to stop infection. Under the current SARS-CoV-2 pandemic, studies of extended viral shedding among children living with HIV are warranted.

To date, there are only a few reports on secondary or superinfections in SARS-CoV-2 patients. A meta-analysis of 28 studies found that the overall bacterial infections in SARS-CoV-2 patients were 7.1%, suggesting that bacterial coinfections are relatively uncommon (30); the bacteriological testing method used was, however, not specified in the majority of the studies (14 studies), and four studies used blood culture, a known poorly sensitive method to detect bacterial infections. These results do not discard a role for pneumococcal superinfections, particularly in PCV-underserved settings, as a report from Spain of five SARS-CoV-2 patients showed coinfection with Streptococcus pneumoniae (31). The findings of these recent studies relative to our study underscore the complexity of extrapolating from established human-adapted viruses with relatively low case fatality rates to a novel zoonotic virus with a relatively high case fatality rate that has been thus far predominately described as a primary viral pneumonitis. Another restriction of using endemic CoV as a surrogate for SARS-CoV-2 is that the tropism of the endemic viruses is mainly the upper respiratory tract while SARS-CoV-2 has propensity for both the upper and lower respiratory tract.

CoVs were normally detected in conjunction with other respiratory viruses in both children living with and those living without HIV, which is similar to previous reports (32). Considering all the respiratory viruses tested in the current set of samples, there were no differences in the frequency of multiple detections in PCV9 recipients (26%) compared to placebo recipients (27%) in both children living with (25% versus 21%) and without (27% versus 30%) HIV (22). In the absence of a control group of non-LRTI children, the relationship between CoV infection and disease cannot, however, be definitively established, and this constitutes a limitation of our study. Actually, a case-control study on the etiology of childhood pneumonia in South Africa and the PERCH study were unable to attribute the detection of endemic CoV as causes of pneumonia requiring hospital admission, since the prevalence of CoV infection was similar in cases and controls (2, 33). The PERCH study was, however, not designed to quantify the importance of coinfections, and caution is needed to directly compare those findings with our observations where interactions with Streptococcus pneumoniae might have led CoV to be associated with pneumonia. Our results suggest that CoV infection likely predisposes to superimposed bacterial infections, as previously shown for other respiratory viruses, so although not directly attributable as the cause of pneumonia, as shown by the previous studies using a single-pathogen model approach, it does not exclude CoVs from being in the causal pathway of the disease. An alternate explanation could be that if, for instance, other respiratory viruses interfere with CoV infection, CoV would appear more frequently in the PCV9 group, even in the absence of a biological interaction with Streptococcus pneumoniae. Our vaccine efficacy point estimates for CoV single infection-associated pneumonia and overall CoV-associated pneumonia were very similar, suggesting an interaction between Streptococcus pneumoniae and CoV in the absence of other viral infections.

In the hypothetical scenario of a perfect vaccine against CoV, it would be interesting to explore this key question on whether there is a biological interaction between CoV and Streptococcus pneumoniae by investigating if the incidence or severity of pneumococcal pneumonia would be affected by the viral vaccine.

A limitation of our study is that the children living with HIV in this cohort were not treated with antiretroviral treatment due to lack of availability for children in the public sector in South Africa 20 years ago, which may have contributed to their clinical course and may differ from that of HIV-infected children currently treated with antiretrovirals. The fact that the study was single-center and that children enrolled into the trial might access health care differently than the general population limits the generalization of the results to other settings.

CoVs are classified into four groups, alpha, beta, gamma, and delta; only alpha and beta CoVs are known to infect humans. The beta group is further composed of A, B, C, and D subgroups (34). While CoV-229E and CoV-NL63 are alpha viruses, CoV-OC43, the most common CoV identified in our study, and CoV-HKU1, together with MERS-CoV, SARS-CoV, and SARS-CoV-2, belong to the beta group (35). Although seroprevalence studies suggest that exposure to the four common CoVs is widespread during childhood and that approximately 90% of adults are seropositive for at least one CoV (36), this humoral immunity may not be cross protective against the novel CoV species. *In vitro* studies have, however, shown that CD4^+^ T cells from SARS-CoV-2-naive donors responded not only to common CoVs but also to SARS-CoV-2, suggesting possible cross-reactivity (37, 38). The role of preexisting SARS-CoV-2 cross-reactive T cells in clinical outcomes remains to be determined.

It has been suggested that infection by SARS-CoV-2 in children causes milder clinical symptoms than in adults (39). In this analysis we show that pneumococcal conjugate vaccine protected children living without HIV against CoV-associated pneumonia hospitalization, and this preventive strategy should be explored in the current pandemic.

## MATERIALS AND METHODS

### Population.

We undertook a *post hoc* analysis of children who participated in an efficacy trial of PCV9 in South Africa as described previously (20, 23). Briefly, 39,836 children were recruited from March 1998 to October 2000 and randomized (1:1) to receive 3 doses (6, 10, and 14 weeks of age) of either PCV9 or placebo, including 2,577 children living with HIV and 37,259 without HIV (23). Hospital-based surveillance for all-cause hospitalization among study participants was undertaken until October 2006. All hospitalized children were evaluated by study doctors and underwent HIV testing according to study protocol (23). Nasopharyngeal aspirates (NPAs) were obtained from children hospitalized with LRTI for identification of select respiratory viruses (20) and archived from February 2000 onward. In the present study, only NPAs collected from 1 February 2000 to 31 January 2002 from children <2 years old admitted with pneumonia diagnosis (defined as presence of alveolar consolidation on chest X-ray, or clinical diagnosis of LRTI without wheeze on chest auscultation, but with rales and/or bronchial breathing) were analyzed. If a child had recurrent pneumonia hospitalizations, only NPAs collected at least 28 days apart or with different viruses detected were included in the analysis.

### Viral testing.

Samples were investigated using immunofluorescence assay for RSV, influenza virus, parainfluenza viruses (PIV) I to III and adenovirus and by nested PCR for human metapneumovirus as described previously (20, 21); real-time reverse transcriptase PCR was later performed to detect four human CoVs, OC43, NL63, HKU1, and 229E, as well as human bocavirus, human rhinovirus (hRV), and WU and KI polyomaviruses using the primers and probes as previously detailed (22). In this analysis, we describe the epidemiology of CoV-associated pneumonia in the study cohort, including exploring differences in hospitalization rates between those who were randomized to receive PCV9 and those who received placebo. We have previously used this randomized placebo-controlled trial as a probe to establish that by protecting against pneumococcal disease, there was also a decrease in hospitalizations for virus-associated pneumonia, specifically influenza virus-, RSV-, human metapneumovirus-, and polyomavirus-associated pneumonia (19–21). The rationale of this approach is that the difference in incidence of any disease between PCV9 recipients and placebo recipients that could potentially be associated with vaccine-serotype pneumococcal infection would indicate a role of vaccine serotypes (40). As an example, we have previously reported that PCV9 recipients had a 45% lower incidence of hospitalization for pneumonia in which influenza virus was identified, and as such we concluded that at least a similar proportion of the influenza-associated pneumonias among the placebo recipients was precipitated by coinfection with PCV9 serotypes (20). This is particularly important considering the lack of a sensitive diagnostic tool to diagnose bacterial pneumonia, including the sensitivity of blood culture being only <5% for diagnosing pneumococcal pneumonia in children (41).

### Statistical analysis.

Regression analyses were performed to compare the prevalence of CoVs and the clinical features between children living with and without HIV. Multiple regressions were controlled for age at hospitalization, detection of a virus previously tested, and year of sample collection. Using the concept of vaccine-probe studies (20), we explored whether there was any association between receipt of PCV9 and the risk of hospitalization for pneumonia associated with CoV infection by estimating vaccine efficacy (VE) based on the formula

$$\text{VE}\;(\text{\%})=\frac{\text{incidence rate in the unvaccinated}-\text{incidence rate in the vaccinated}}{\text{incidence rate in the unvaccinated}}\times 100$$

All randomized children were included in the intent-to-treat analysis from the day they received their first dose of study vaccine. The first episode was included in the VE calculation for an individual participant, and *P* values of ≤0.05 were considered significant. Analyses were performed using STATA version 13.1 (College Station, TX, USA).

### Ethical considerations.

The main efficacy trial and subsequent *post hoc* analyses were approved by the Human Research Ethics Committee (Medical) of the University of the Witwatersrand. Signed written informed consent was obtained from the parents/legal guardians as part of the trial. The Ethics Committee did not require additional consent for this analysis. The main study was not registered under any clinical trial registry as it was undertaken prior to registration being made mandatory.
